# Molecular subtypes based on cuproptosis-related genes and immune profiles in lung adenocarcinoma

**DOI:** 10.3389/fgene.2022.1006938

**Published:** 2022-10-12

**Authors:** Yufei Wang, Chen Zhang, Chengyue Ji, Wenli Jin, Xin He, Shunzhi Yu, Renhua Guo

**Affiliations:** ^1^ Zhejiang University-University of Edinburgh Institute, Zhejiang University, Haining, Zhejiang, China; ^2^ Jiangsu Province Hospital and Nanjing Medical University First Affiliated Hospital, Nanjing, Jiangsu, China; ^3^ Shanghai Tenth People’s Hospital, Tongji University School of Medicine, Shanghai, China

**Keywords:** cuproptosis, LUAD, molecular subtypes, prognosis, tumor microenvironment, immune infiltration

## Abstract

**Background:** Recent studies have identified several molecular subtypes of lung adenocarcinoma (LUAD) that have different prognoses to help predict the efficacy of immunotherapy. However, the prognostic prediction is less than satisfactory. Alterations in intracellular copper levels may affect the tumor immune microenvironment and are linked to cancer progression. Previous studies have identified some genes related to cuproptosis. The characteristics of the cuproptosis molecular subtypes have not been thoroughly studied in LUAD.

**Methods:** The transcriptomic data and clinical information of 632 LUAD patients were used to investigate the LUAD molecular subtypes that are associated with the cuproptosis-related genes (CRGs), the tumor immune microenvironment, and stemness. The cuproptosis score was constructed using univariate Cox regression and the minor absolute shrinkage and selection operator (LASSO) to quantify the prognostic characteristics.

**Results:** Three different molecular subtypes related to cuproptosis, with different prognoses, were identified in LUAD. Cluster A had the highest cuproptosis score and the worst prognosis. Patients in the high cuproptosis score group had a higher somatic mutation frequency and stemness scores. Patients in the low cuproptosis score group had more immune infiltration and better prognosis.

**Conclusion:** Molecular subtypes of LUAD based on CRGs reflect the differences in LUAD patients. The cuproptosis score can be used as a promising biomarker, which is of great significance to distinguish the relationship between cuproptosis and the immune microenvironment. The cuproptosis signature based on the cuproptosis score and clinical characteristics of individual patients will be useful for guiding immunotherapy in LUAD.

## 1 Introduction

With an estimated 2 million new cases and 1.76 million deaths each year, lung cancer is one of the most commonly diagnosed cancers and the leading cause of cancer-related deaths worldwide. Lung adenocarcinoma (LUAD) is the most common histological type of lung cancer (Thai et al., 2021).

In recent years, immune checkpoint inhibitors alone or in combination with chemotherapy have significantly improved survival in patients with advanced LUAD. In the KEYNOTE-024 study, patients without EGFR/ALK aberrations treated with pembrolizumab had significantly improved overall survival (30.0 months; 95% CI, 18.3 months to not reached) compared with chemotherapy (14.2 months; 95% CI: 9.8–19.0 months) (HR, 0.63; 95% CI, 0.47–0.86) (Reck et al., 2019). In the KEYNOTE-189 study, the overall survival (22.0 months; 95% CI, 19.5–25.2 months) for patients treated with pembrolizumab in combination with chemotherapy in nonsquamous non-small cell lung cancer was significantly longer than that in the placebo group (HR, 0.56; 95% CI, 0.45–0.70) (Gadgeel et al., 2020).

Although immunotherapy is an effective treatment, not all patients with LUAD can benefit from it. In addition, LUAD is a heterogeneous disease, which also makes identifying new subtypes essential to predicting prognosis and ensuring that patients receive personalized treatment (Liu et al., 2021). Recently, an increasing number of molecular subtypes have been studied to predict the efficacy of immunotherapy. For example, Zhang et al. isolated three types of ferroptosis-related molecules in LUAD, which helped predict the prognosis, tumor microenvironment (TME) cell infiltration characteristics, and immunotherapy effects in patients with LUAD (Zhang et al., 2021). Wang et al. identified two distinct subtypes of LUAD. The high-risk subtype was characterized by a lower TIDE score, increased programmed death-ligand 1 (PD-L1) expression, higher tumor mutation burden (TMB), elevated levels of the cell cycle modulators CDK4/CDK6, and TP53 mutations, and it was implicated for immune checkpoint blockade therapy (Wang et al., 2020). Wu et al. (2021) established a promising immunoprognostic model associated with TP53 to identify early-stage LUAD patients with a high risk of unfavorable survival. These studies have shed light on the molecular subtypes of LUAD. However, the above classification strategies for predicting immune efficacy were not sufficiently effective. Thus, finding new molecular subtypes of LUAD is vital for identifying potential benefits of immunotherapy.

Copper-induced proptosis (cuproptosis) is a novel form of cell death induced by excessive intracellular copper (Tang et al., 2022). Copper overload induces lipoylated dihydrolipoamide S-acetyltransferase (DLAT) aggregation, which is associated with mitochondrial tricarboxylic acid (TCA) cycle activation. This results in proteotoxic stress and leads to cuproptosis (Wang et al., 2022). In recent studies, alterations in intracellular copper levels have been linked to cancer development and progression, including lung cancer (Ge et al., 2022). Therefore, cuproptosis may serve as a novel target for treating LUAD. Recent studies have shown that intracellular copper regulates key signaling pathways mediating PD-L1-driven cancer immune evasion (Voli et al., 2020). These findings suggest that cuproptosis may affect the tumor immune microenvironment, and identification of the characteristics of cuproptosis may effectively predict the efficacy of immunotherapy.

Previous studies have identified certain genes related to cuproptosis (Polishchuk et al., 2019; Aubert et al., 2020; Dong et al., 2021; Ren et al., 2021; Bian et al., 2022; Kahlson and Dixon, 2022; Tang et al., 2022; Tsvetkov et al., 2022). Several studies have reported the predictive value of these cuproptosis-related genes (CRGs) in LUAD. Li et al. constructed a prognostic model for patients with radiotherapy resistance based on CRGs screened from RNA-sequencing data of radiation-treated cell lines (Li et al., 2022). Hu et al. 2022 and Zhang et al. (2022) also used different CRGs to construct risk models to predict the prognosis of LUAD patients. However, the role of CRGs in affecting the immune microenvironment in LUAD has yet to be explored. In the present study, 632 LUAD samples were divided into three cuproptosis-related subtypes based on differentially expressed genes (DEGs) of LUAD subtypes according to CRGs and immune profiles. Additionally, a model with cuproptosis scores was established. The characteristics of the immune microenvironment between low and high cuproptosis score groups were explored. These findings show that the cuproptosis score might be an independent prognostic factor for LUAD patients and predict the clinical efficacy of immunotherapy.

## 2 Materials and methods

### 2.1 Data sources and preprocessing

The transcriptomic data and clinical information of LUAD patients were downloaded from the TCGA database (https://portal.gdc.cancer.gov/) and Gene Expression Omnibus (GEO) database (https://www.ncbi.nlm.nih.gov/geo/). Patients of different sexes and races were included, and patients without survival information were excluded. The transcriptomic data and clinical information of 632 tumor samples were collected. Of these tumor samples, 516 were from the TCGA-LUAD dataset and 116 were from GEO (GSE26939) (Wilkerson et al., 2012). Additionally, information on 59 normal samples was collected from TCGA. The mutation information of 557 LUAD patients was obtained from the TCGA database. The GDC GISTIC copy number (gene-level) dataset of 531 LUAD patients was obtained from UCSC Xena (https://xena.ucsc.edu/). All transcriptomic data were processed to log2 form. All gene expression levels that repeatedly appeared in multiple rows were averaged and kept in one row. All data were downloaded in June 2022. The R (version 4.2.0) and R Bioconductor packages were used for all data analyses.

### 2.2 Identification of CRGs in LUAD

Initially, 19 cuproptosis-related genes (*NFE2L2, NLRP3, ATP7B, ATP7A, SLC31A1, FDX1, LIAS, LIPT1, LIPT2, DLD, DLAT, PDHA1, PDHB, MTF1, GLS, CDKN2A, DBT, GCSH,* and *DLST*) were identified from previous studies (Polishchuk et al., 2019; Aubert et al., 2020; Dong et al., 2021; Ren et al., 2021; Bian et al., 2022; Kahlson and Dixon, 2022; Tang et al., 2022; Tsvetkov et al., 2022). Cuproptosis-related gene expression was determined in normal cells and LUAD tumor cells from the TCGA-LUAD dataset. The difference between the normal and tumor groups was analyzed using the Wilcoxon test and displayed in a box plot. *p* values less than 0.05 were considered to indicate significant differences in gene ontology.

### 2.3 Detection of CRG-related mutations in LUAD

The numbers of mutated genes were calculated using the mutation information of each sample obtained from the TCGA database. The mutation information of 19 CRGs and the clinicopathological characteristics were displayed in a waterfall plot using the R package “maftools”. The copy number variation (CNV) frequencies of 19 CRGs are displayed in a bar plot. The “RCircos” package in R was applied to show the location of the 19 CRGs on chromosomes.

### 2.4 Survival analysis of CRGs in LUAD patients

The expression of the CRGs was extracted from 632 merged data from TCGA-LUAD and GSE26939, and 17 of these CRGs (N*FE2L2, ATP7B, ATP7A, SLC31A1, FDX1, LIAS, LIPT1, DLD, DLAT, PDHA1, PDHB, MTF1, GLS, CDKN2A, DBT, GCSH,* and *DLST*) were extracted. The 95% confidence interval (CI), hazard ratios (HR), and *p* values of these 17 CRGs were calculated by univariate Cox regression using the “Survival” package in R. The interaction and impact of each CRG on the prognosis are shown in a network.

### 2.5 Correlation analysis of CRGs and LUAD immune estimation

The ESTIMATE tool from the R package “estimate” and gene expression signatures were used to estimate the fraction of stromal and immune cells within the tumor samples and to estimate the elements of the TME, including StromalScore, ImmuneScore, ESTIMATEScore, and TumorPurity (Yoshihara et al., 2013).

CIBERSORT, a method that reduces noise and unidentified mixtures, can recognize the composition of tumor cells by using gene expression profiles (Newman et al., 2015). Correlations between the CIBERSORT data for immune cell infiltration and the 4 CRGs (L*IPT1, DLAT, PDHA1,* and *DLST*) that were significantly associated with survival situations are displayed. Additionally, correlation tests between these 4 CRGs and the ImmuneScore were performed using Spearman analyses.

### 2.6 Clustering of LUAD patients based on CRGs

The R package “ConsensusClusterPlus” was used for consensus clustering and result visualization (Wilkerson and Hayes, 2010). The clustering was based on the 2 CRGs (*PDHA1* and *DLAT*) with the two highest correlation scores, as determined by ImmuneScore. The efficacy of the consensus clustering was determined by performing principal component analysis (PCA). Two cuproptosis subtypes (CRG cluster A and CRG cluster B) were found. Clinical characteristics based on the cuproptosis subtypes are displayed in a heatmap.

Functional and pathway enrichment analyses of the cuproptosis subtypes were performed. The “GSEABase” and “GSVA” R packages were applied for pathway enrichment analysis and to analyze the differences in biological functions among the different cuproptosis clusters (Hänzelmann et al., 2013). The gene sets of “c2. cp.kegg.symbol” and “c5. go.symbols” were downloaded from Gene Set Enrichment Analysis (GSEA) (https://www.gsea-msigdb.org/gsea/datasets.jsp) and were used to run GSVA enrichment analysis.

The single sample gene set enrichment analysis (ssGSEA) method was used to evaluate the scores of the TME cells in each LUAD sample (Barbie et al., 2009). The immune infiltration of the cuproptosis subtypes is displayed in a box plot.

### 2.7 Clustering of LUAD patients based on the DEGs between the cuproptosis subtypes

Using the linear model and empirical Bayes statistics for differential expression in the R package “limma,” 122 DEGs between the two cuproptosis subtypes were identified (absolute logFC >0.585 and adjusted *p* values <0.05). Gene Ontology (GO) and Kyoto Encyclopedia of Genes and Genomes (KEGG) were used as references, and enrichment analysis of DEGs was performed by the R package “clusterProfiler.” With univariate Cox regression analysis, 72 DEGs (*p* values less than 0.05) were found, which were then used for consensus clustering. PCA was performed to check the efficacy of the consensus clustering. Three gene subtypes (geneCluster A, geneCluster B, and geneCluster C) were found. The clinical characteristics based on gene subtypes are displayed in a heatmap. CRG expression differences between gene subtypes were analyzed using the Wilcoxon test and are shown in a box plot.

### 2.8 Construction of the cuproptosis prognostic model

The samples were divided into training and testing sets randomly and kept the numbers of the training and testing sets were the same. The samples in the training set were used to construct the model, and the samples in the testing set were used to verify the accuracy of the model. The most powerful prognostic genes among the DEGs were identified using the univariate Cox regression model with the minor absolute shrinkage and selection operator (LASSO) (Gao et al., 2010). Finally, eight genes and their correlative coefficients were obtained to construct the cuproptosis gene signature. These genes were *DLST* (coefficient = 0.268908425), *PDHA1* (coefficient = 0.320688231), *FOXM1* (coefficient = 0.152692216), *EN O 3* (coefficient = −0.249542466), *CD79A* (coefficient = −0.108306171), *AMBP* (coefficient = −0.117611131), *CPS1* (coefficient = 0.121531233), and *NTS* (coefficient = 0.101175652). The cuproptosis score was defined as the sum of each gene’s expression × correlative coefficient. LUAD samples were divided into high and low cuproptosis score groups using the median cuproptosis score 0.8963089 as the boundary. Survival analysis was performed for the two groups using the R package “survminer.” Receiver operating characteristic (ROC) curve analysis was conducted using the R package “timeROC” to obtain the area under the curve (AUC) value and evaluate the predictive power of the signature. A regression nomogram of the cuproptosis score and other clinical covariates of LUAD patients was constructed using the R package “regplot.” The calibration curves of the cuproptosis score and other clinical covariates of LUAD patients were estimated using the R package “rms.”

### 2.9 Analysis of the immune microenvironment and stemness of tumors in different cuproptosis score groups

The mutation information of LUAD patients was divided into low and high cuproptosis score groups based on cuproptosis scores. Waterfall plots for the mutation information of the 20 genes with the highest mutation frequency were plotted for the low and high cuproptosis score groups. The immune infiltration of low and high cuproptosis score groups was displayed with the scores of TME cells in each LUAD sample evaluated using the ssGSEA algorithm. The ESTIMATE scores, including the StromalScore, ImmuneScore, and ESTIMATEScore, of the low and high cuproptosis score groups are displayed in a violin plot. Additionally, the correlation between the stemness score (RNAss) and the cuproptosis score was calculated and plotted.

### 2.10 Validation of cuproptosis prognostic model using two LUAD immunotherapy cohorts

Transcriptomic data and clinical information of 34 NSCLC patients treated with anti-programmed death-1 (PD-1) therapy were collected from Tumor Immunotherapy Gene Expression Resource (http://tiger.canceromics.org/). Dataset IDs are NSCLC_GSE126044 (Cho et al., 2020) and NSCLC_GSE135222 (Jung et al., 2019). Samples that had no information about the immunotherapy response were omitted, and 34 samples were finally included. The cuproptosis scores were calculated. These patients were divided into high and low cuproptosis score groups and were checked with immunotherapy responses.

## 3 Results

### 3.1 Identification and characteristics of CRGs involved in LUAD progression

**FIGURE 1:**
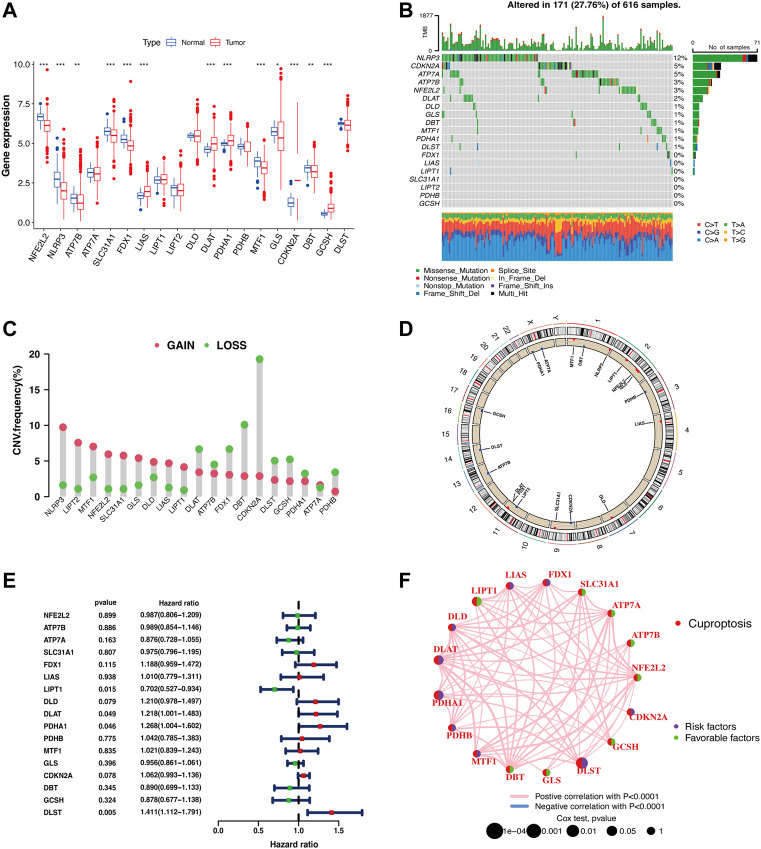
Identification of CRGs and detection of mutations in LUAD. **(A)** Cuproptosis-related gene expression in normal cells and LUAD tumor cells (Wilcoxon test; *p < 0.05; **p < 0.01; ***p < 0.001). **(B)** The waterfall plot displays the mutation frequency of CRGs in LUAD samples. **(C)** CNV of CRGs in LUAD samples. **(D)** CNV locations of CRGs are labeled on the chromosome (red dots: CNV gain frequency is higher; blue dots: CNV loss frequency is higher). **(E)** Forest plot for the univariate Cox regression analysis of CRGs associated with clinical prognosis in LUAD patients. **(F)** Prognostic correlation between CRGs.

A LUAD cohort including 59 normal samples and 539 tumor samples from TCGA was included in this study. Most of the CRG expression levels were significantly different between the normal and tumor groups (Figure 1A). Twelve out of the 19 CRGs had a mutation frequency >1% and were closely associated with progression or recurrence in LUAD (Figure 1B). In addition, we detected widespread CNVs in these CRGs (Figure 1C). *NLRP3, LIPT2, MTF1, NFE2L2, SLC31A1, GLS, DLD, LIAS, LIPT1,* and *ATP7A* showed extensive CNV amplification, whereas *ATP7B, FDX1, DLAT, PDHA1, PDHB, CDKN2A, DBT, GCSH,* and *DLST* showed copy number loss. The chromosomal locations of the CRGs with CNVs are shown in Figure 1D. Univariate Cox regression was used to explore the relationship between the CRGs and clinical prognosis in LUAD patients, and the results showed that *LIPT1* (HR = 0.702 (0.527–0.934), *p* = 0.015), *DLAT* (HR = 1.218 (1.001–1.483), *p* = 0.049), *PDHA1* (HR = 1.268 (1.004–1.602), *p* = 0.046), and *DLST* (HR = 1.411 (1.112–1.791), *p* = 0.005) could be relevant to the prognostic situation (Figure 1E). Next, the CRGs were constructed into a network map, which enabled a comprehensive analysis of the interactions and interconnections of the genes and their impact on the prognosis of patients with LUAD. The interconnections between these genes were all positive correlations. *DLST, DLAT,* and *PDHA1* were major risk factors, while *LIPT1* was a favorable factor (Figure 1F).

### 3.2 Identification of CRGs associated with LUAD immune profiles

**FIGURE 2:**
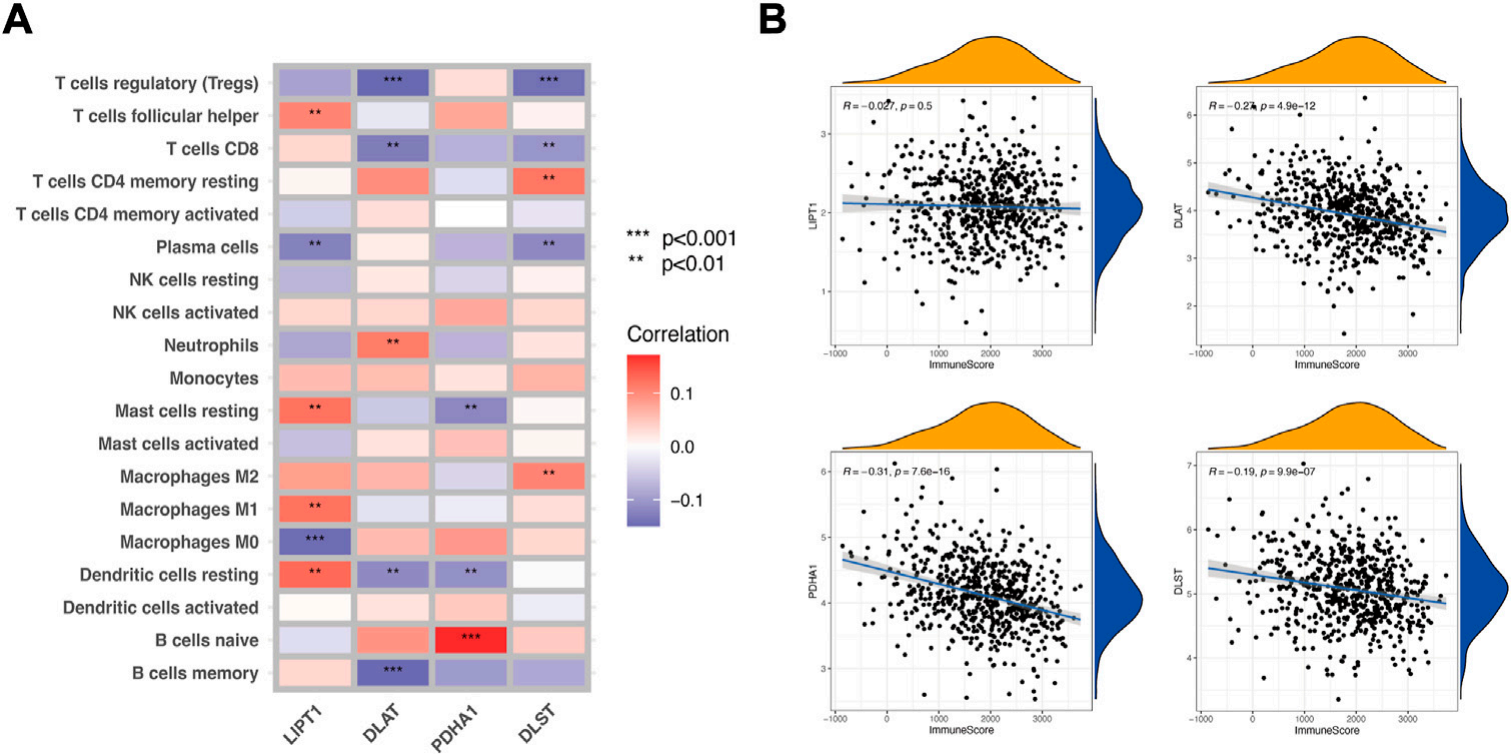
Identification of CRGs associated with LUAD immune profiles. **(A)** Correlation between the CIBERSORT data of immune cell infiltration and the 4 CRGs (*LIPT1*, *DLAT, PDHA1,* and *DLST*). **(B)** Correlation between the ImmuneScore of ESTIMATE and the 4 CRGs.

To explore whether the expression of CRGs impacted LUAD immune profiles, we extracted the expression of the four CRGs associated with prognosis and applied the ESTIMATE tool and CIBERSORT algorithm to calculate the ESTIMATE scores and immune cell infiltration of LUAD patients. As shown in Figure 2A, *LIPT1* had a positive correlation (*p* < 0.01) with follicular helper T cells, resting mast cells, M1 macrophages, and resting dendritic cells, while a negative correlation (*p* < 0.01) with plasma cells and M0 macrophages. *DLAT* had a positive correlation (*p* < 0.01) with neutrophils, while a negative correlation (*p* < 0.01) with regulatory T cells (Tregs), CD8^+^ T cells, resting dendritic cells, and memory B cells. *PDHA1* had a positive correlation (*p* < 0.001) with naïve B cells while a negative correlation (*p* < 0.01) with resting mast cells and resting dendritic cells. *DLST* had positive correlation (*p* < 0.01) with memory CD4^+^ T cells and M2 macrophages, while negative correlation (*p* < 0.01) with Tregs, CD8^+^ T cells and plasma cells. We further analyzed the correlation between the 4 CRGs and the ImmuneScore. The results show that three CRGs (*DLAT*, *PDHA1,* and *DLST*) were significantly correlated with the ImmuneScore (Figure 2B, *p* < 0.001), although the correlation coefficient was slightly weak. We selected the first two CRGs with the highest absolute coefficients, *PDHA1* (R = .0.31, *p* < 0.001) and *DLAT* (R = .0.27, *p* < 0.001) (Figure 2B), to construct an immune-associated signature.

### 3.3 Cuproptosis subtypes in LUAD

**FIGURE 3:**
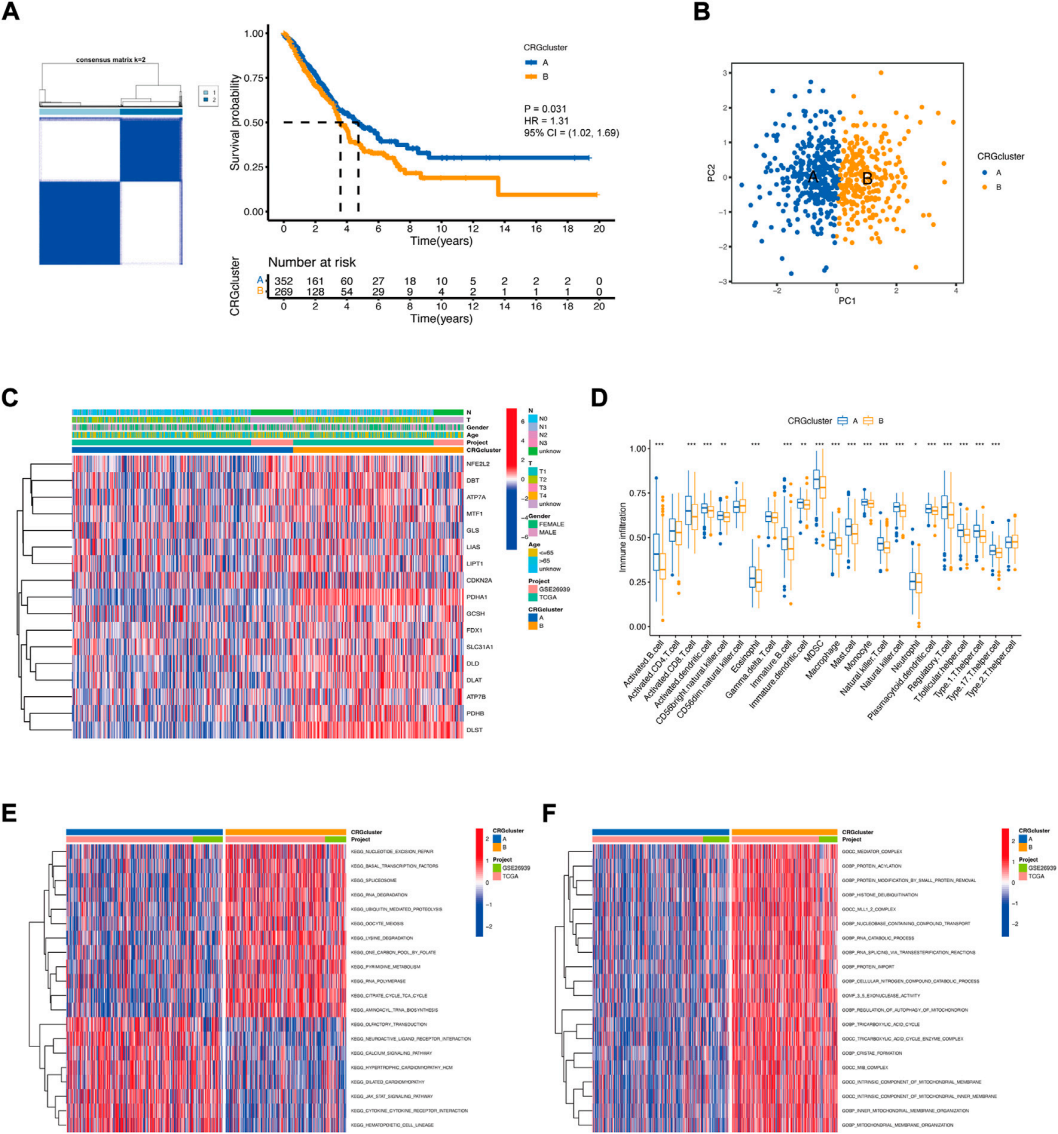
The landscape of the cuproptosis and the biological characteristics of the cuproptosis subtypes in LUAD. **(A)** Clustering of patients with LUAD based on PDHA1 and DLAT expression. Consensus clustering matrix for k = 2. Kaplan−Meier plot of the CRG clusters and overall survival probability. **(B)** PCA of two cuproptosis subtypes. **(C)** Relationship between the cuproptosis subtypes and various clinical characteristics. **(D)** TME-infiltrating cell composition between the two cuproptosis subtypes (Wilcoxon test; *p < 0.05; **p < 0.01; ***p < 0.001). **(E,F)** Functional and biological differences between the cuproptosis subtypes.

We extracted *PDHA1* and *DLAT* expression data from LUAD patients and performed consensus clustering. Two clusters of patients were found. There were 352 patients in Cluster A and 269 patients in Cluster B. Survival analysis revealed that prognosis differed substantially among these two subtypes, and Cluster A had considerable survival advantages (*p* = 0.031) (Figure 3A). PCA further confirmed two remarkably different subtypes (Figure 3B). The relationship between the two subtypes and various clinical characteristics was studied, which also confirmed two remarkably different subtypes. Most CRGs were highly expressed in Cluster B (Figure 3C). Next, the immune infiltration score of the 23 types of immune cells was evaluated in the two subtypes using ssGSEA (Figure 3D). In Cluster A, the most significant immune-infiltrating cells were activated B cells, activated CD8^+^ T cells, activated dendritic cells, macrophages, and natural killer cells. Most of the immune cells showed less infiltration in Cluster B (Figure 3D). This may also indicate that Cluster A had better immunity and prognosis. Then, GSVA enrichment analysis was performed on the two subtypes to examine their functional and biological differences (Figures 3E,F). The results showed that the two subtypes differed significantly with respect to metabolic pathways, such as the pyrimidine metabolism pathway and the citrate cycle (TCA cycle) pathway. These findings suggest that metabolic alterations may contribute to the distinct cuproptosis subtypes. Additionally, some pathways of biological functions significantly differed between the subtypes, such as the protein acylation pathway, histone deubiquitination pathway, and protein import pathway. These pathways were highly activated in Cluster B and contributed to poor prognosis.

**FIGURE 4:**
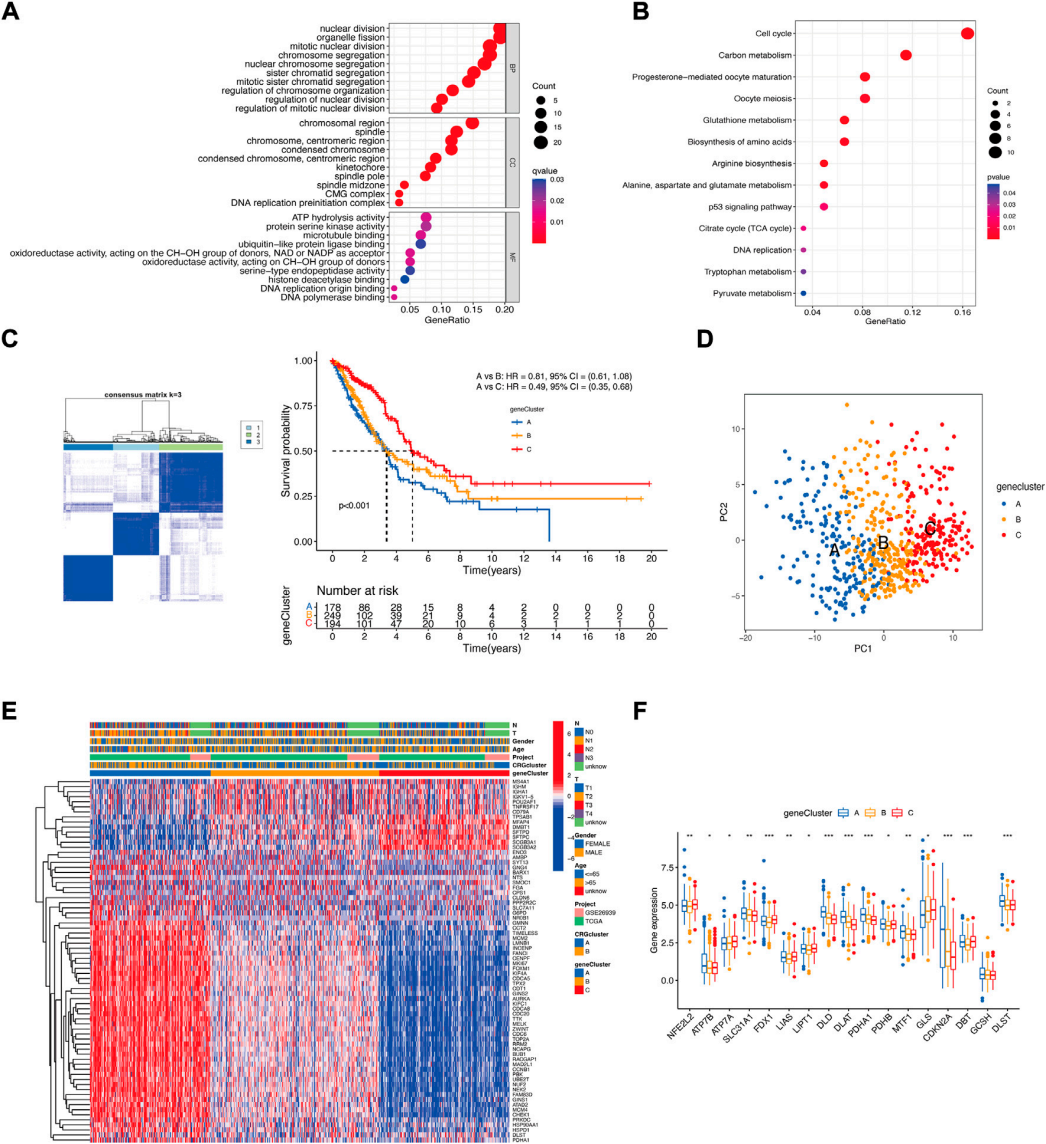
The landscape of the biological characteristics of cuproptosis gene cluster. **(A)** GO enrichment analysis of DEGs. BP: biological process, CC: cellular component, MF: molecular function. **(B)** KEGG enrichment analysis of DEGs. **(C)** Clustering of LUAD patients based on DEGs associated with the cuproptosis subtypes. Consensus clustering matrix for k = 3. Kaplan−Meier plot of the gene clusters and overall survival probability. **(D)** PCA of three gene clusters. **(E)** Clinical characteristics of the three gene clusters. **(F)** CRG expression differences among the three gene clusters (Kruskal–Wallis test; *p < 0.05; **p < 0.01; ***p < 0.001).

To further study the potential biological function of cuproptosis clusters in LUAD, differential expression analysis was performed, and 122 DEGs were identified. GO enrichment analysis showed that the cuproptosis-related DEGs were considerably enriched in the process of mitotic division (Figure 4A). KEGG enrichment analysis showed that cuproptosis-related DEGs were considerably enriched in some metabolic pathways, such as carbon metabolism, glutathione metabolism, amino acid biosynthesis, and the TCA cycle. These enrichments could indicate the specific metabolic demands of cuproptosis, which is an idea that is supported by emerging literature on the participation of lipoylated TCA cycle proteins in cuproptosis (Li et al., 2022).

Then, we performed univariate Cox regression analysis, and 72 DEGs were used for consensus clustering. Three clusters of patients were found. There were 178 patients in Cluster A, 249 patients in Cluster B, and 194 patients in Cluster C. Survival analysis revealed that prognosis differed substantially among these three subtypes, and Cluster C had considerable survival advantages (*p* < 0.001) (Figure 4C). The three subtypes could be significantly separated on the basis of the expression levels of the DEGs (Figure 4D). The relationship between the three subtypes and various clinical characteristics was studied and is shown in a heatmap (Figure 4E). Next, CRG expression was evaluated in these three subtypes. As expected, there were significant differences in the expression of the CRGs among the three gene clusters (Figure 4F).

### 3.4 Construction of a prognostic cuproptosis signature

**FIGURE 5:**
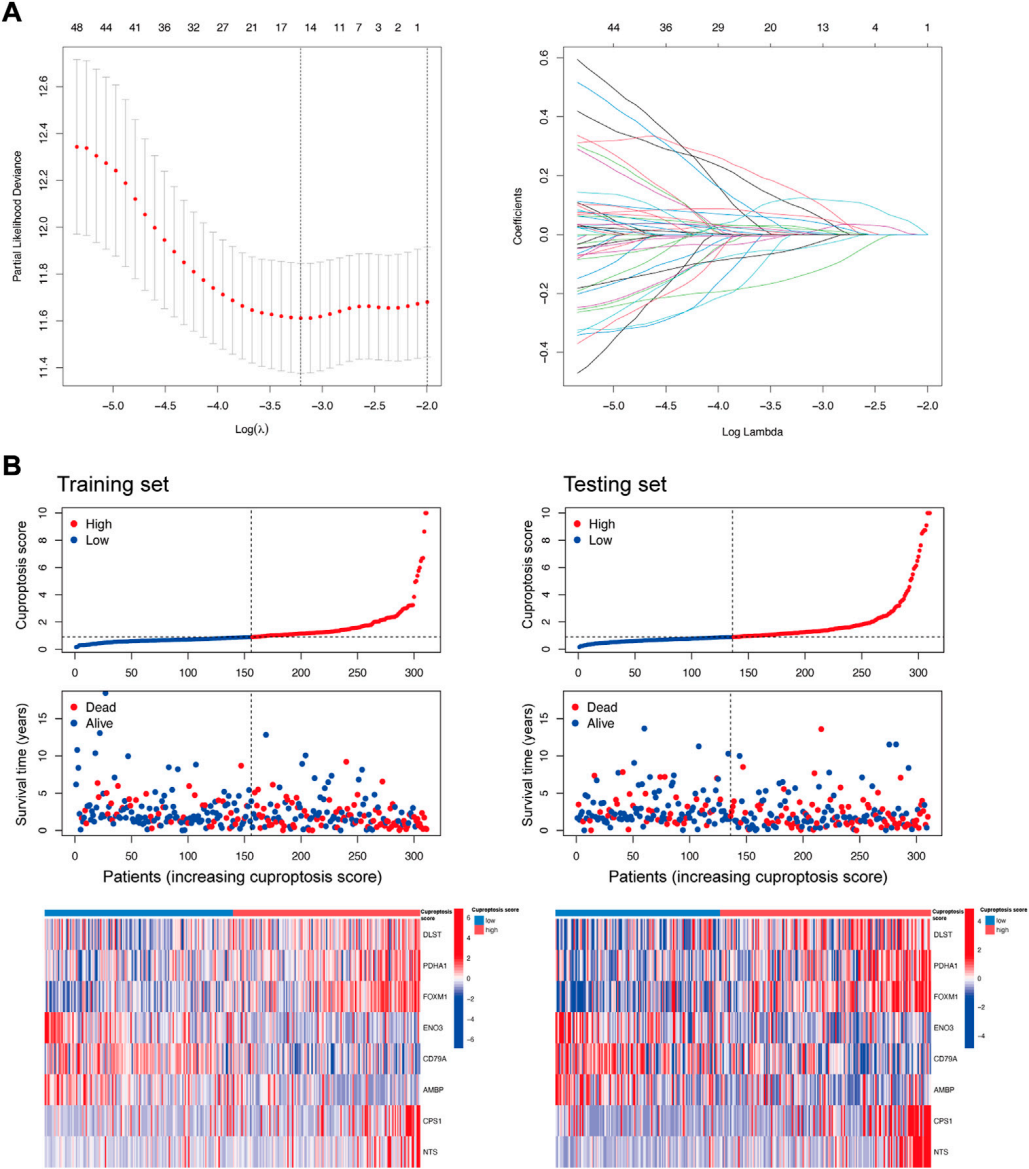
DEGs selection for the construction of the cuproptosis prognostic model. **(A)** Partial likelihood deviance and coefficients for LASSO. **(B)** Distribution of the cuproptosis score, survival status, and the expression of DEGs (*DLST, PDHA1, FOXM1, ENO3, CD79A, AMBP, CPS1,* and *NTS*) for modeling in the training set and testing set.

To further understand the characteristics of cuproptosis in each patient with LUAD, we performed univariate Cox regression and LASSO to select the 8 most powerful prognostic genes among the DEGs (Figure 5A). A higher cuproptosis score was associated with increased death and decreased survival time both in the training set and the testing set. The expression distribution of DEGs for modeling was different in the low and high cuproptosis score groups (Figure 5B).

**FIGURE 6:**
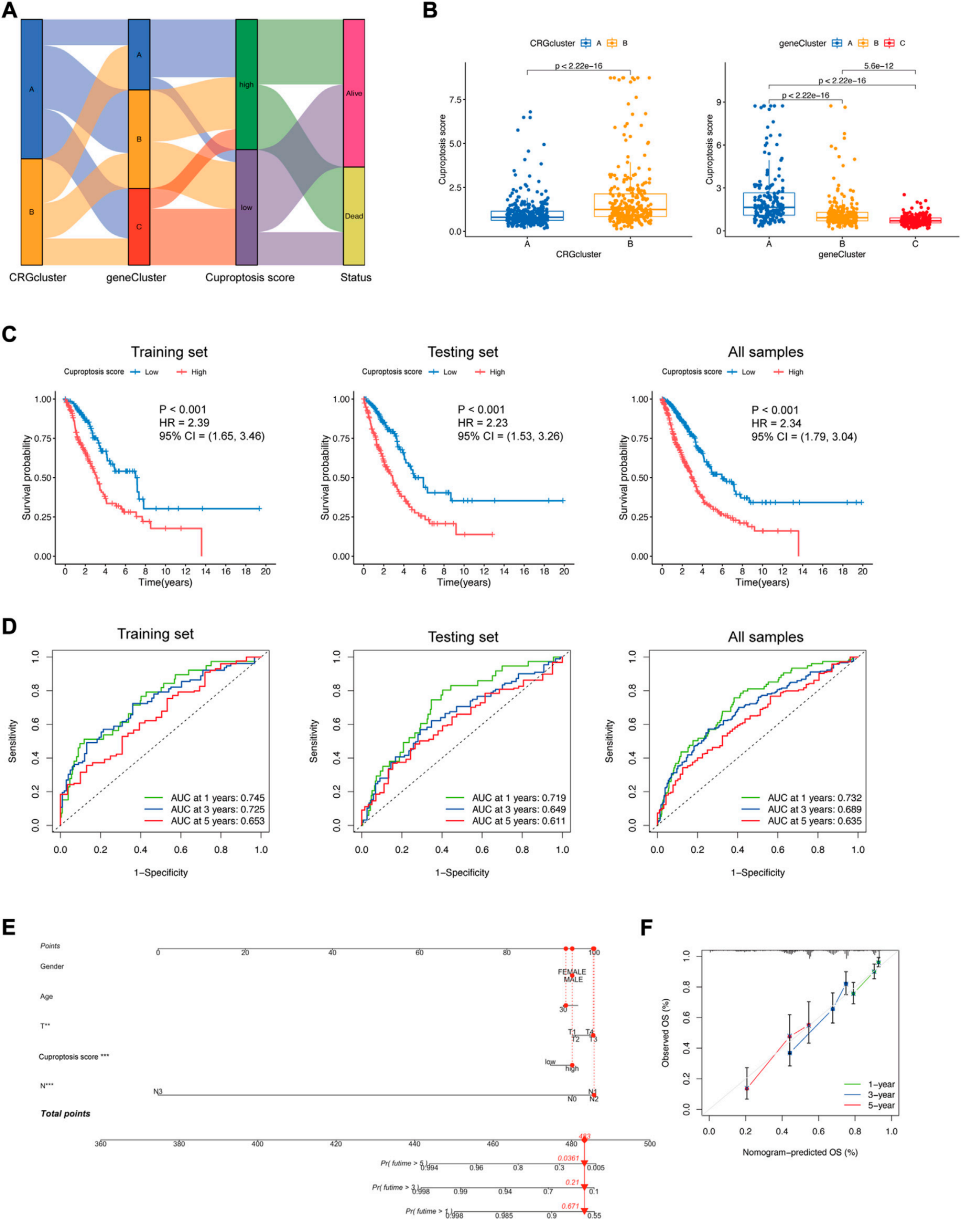
Construction of the cuproptosis signature. **(A)** Sankey diagram displaying the relationships among CRG-related clusters, DEG-related clusters, the cuproptosis score, and the status of LUAD patients. **(B)** Cuproptosis score differences in CRG-related clusters and DEG-related clusters (Wilcoxon test). **(C)** Kaplan−Meier plots of the cuproptosis signature and overall survival probability in the training set, testing set, and all samples. **(D)** ROCs for one-year, three-year, and five-year survival prediction. **(E)** Nomogram model in LUAD patients. The cuproptosis score is an independent prognostic factor. **(F)** Calibration curve for the overall survival (OS) of the nomogram model. A dashed diagonal line represents the ideal nomogram.

A Sankey diagram demonstrated the association between clusters, cuproptosis score, and clinical status. Most LUAD patients in DEG-related Cluster A had a high cuproptosis score. Most LUAD patients in DEG-related Cluster C had a low cuproptosis score. Additionally, the cuproptosis score influenced the status of the patients (Figure 6A). The cuproptosis score was significantly higher in CRG-related Cluster B and DEG-related Cluster A (Figure 6B), which is consistent with the former result that CRG-related Cluster B and DEG-related Cluster A had a poor prognosis (Figures 3A,4C). Thus, the cuproptosis score could be considered a prognostic factor of LUAD.

To further explore the prognostic difference between the low and high cuproptosis score groups, survival analysis and ROC curve analysis were performed to evaluate the predictive power of the cuproptosis signature. In the training set, testing set, and all sample groups, patients with a higher cuproptosis score had a significantly shorter survival time (*p* < 0.001) (Figure 6C). The AUCs of the 1-year, 3-year, and 5-year ROC curves were all between 0.6 and 0.8 (0.745, 0.725, and 0.653 for the training set; 0.719, 0.649, and 0.611 for the testing set; 0.732, 0.689, and 0.635 for all samples), which represented fair model quality (Figure 6D).

To facilitate the clinical application of the prediction model, we integrated clinical information (sex, age, T stage, and N stage) and gene features of LUAD patients and performed the multivariable Cox regression analysis model to develop the nomogram (Figure 6E). Calibration curves demonstrated favorable concordance between the predicted OS and the observed OS at 1, 3, and 5 years of survival (Figure 6F), reflecting a relatively excellent predictive performance of the nomogram. The cuproptosis score could be used as an independent prognostic factor.

### 3.5 Characteristics of the cuproptosis signature

**FIGURE 7:**
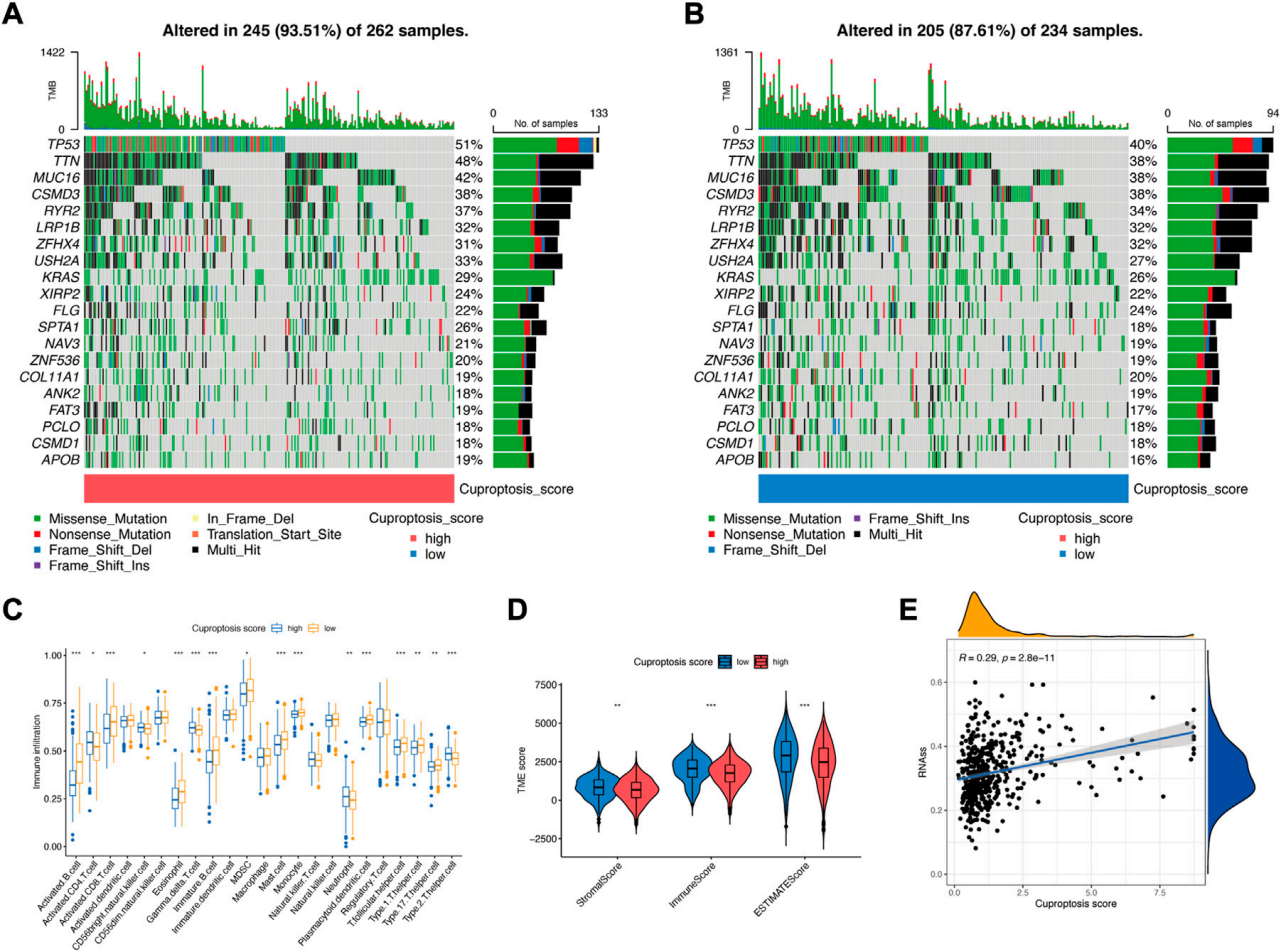
Characteristics of the cuproptosis signature. **(A)** Somatic mutations in the high cuproptosis score group. **(B)** Somatic mutations in the low cuproptosis score group. **(C)** TME-infiltrating cell composition between high and low cuproptosis score groups (Wilcoxon test; *p < 0.05; **p < 0.01; ***p < 0.001). **(D)** ESTIMATE score in the low and high cuproptosis score groups (Wilcoxon test; *p < 0.05; **p < 0.01; ***p < 0.001). **(E)** Spearman correlation analysis between the stemness score (RNAss) and cuproptosis score.

Somatic mutations were compared in LUAD patients with low and high cuproptosis scores, and the top 20 genes with the highest mutation frequency were visualized. The total mutation frequencies of these top 20 genes were relatively higher in patients with high cuproptosis scores (93.51%) than in those with low cuproptosis scores (87.62%) (Figures 7A,B). Fisher’s exact test showed that the mutation frequencies of *TP53* (*p* = 0.015), *TTN* (*p* = 0.029), and *SPTA1* (*p* = 0.039) were significantly different between these two groups (Supplementary Table S1). However, the univariate Cox regression showed that none of these three gene mutations affected the prognosis (Supplementary Figure S1). The immune infiltration score of the 23 types of immune cells was evaluated in low and high cuproptosis score groups. Activated B cells, activated CD8^+^ T cells, activated dendritic cells, and T helper cells were significantly higher in the low cuproptosis score group (Figure 7C), which indicates that a low cuproptosis score may be related to strengthened antitumor immunity. The StromalScore, ImmuneScore, and ESTIMATEScore were all significantly lower in the high cuproptosis score group (Figure 7D), which indicates that tumors in the high cuproptosis score group were purer, more aggressive, and had a worse prognosis. Additionally, the stemness score (RNAss) was positively correlated with the cuproptosis score (R = 0.29, *p* < 0.001) (Figure 7E). This finding indicates stronger heterogeneity and a worse prognosis in the high cuproptosis score group.

### 3.6 Relationship between the cuproptosis score and the effect of immunotherapy

**FIGURE 8:**
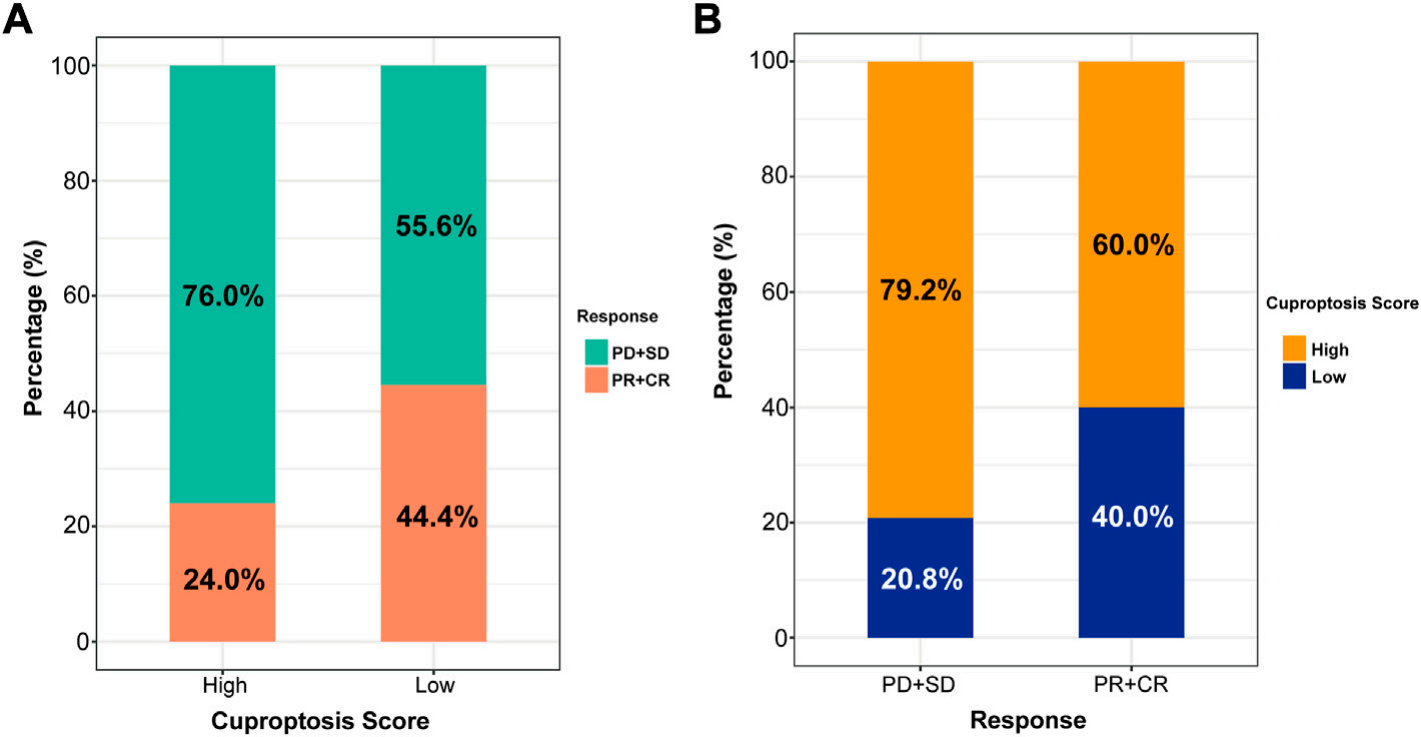
The role of cuproptosis score in anti-PD-1 immunotherapy. **(A)** Proportions of anti-PD-1 immunotherapy response in high and low cuproptosis score groups. PR, partial response, PD, progressive disease, SD, stable disease, CR, complete response. **(B)** Proportions of patients with high and low cuproptosis scores in different anti-PD-1 immunotherapy responses.

At present, anti-PD-1 therapy plays an important role in LUAD immunotherapy. To further illustrate the relationship between the cuproptosis score and the efficacy of immunotherapy, two LUAD anti-PD-1 treatment cohorts were investigated. The patients were divided into high and low cuproptosis score groups. As shown in Figure 8A, the high cuproptosis score group had a lower objective response rate [ORR, the percent of patients with complete response (CR) and partial response (PR), 24.0% vs. 44.4%, *p* = 0.395]. In addition, the rate of patients with high cuproptosis scores was higher in the nonresponder [defined as patients with progressive disease (PD) and stable disease (SD), 79.2% vs. 60.0%, *p* = 0.395, Figure 8B].

## 4 Discussion

In recent years, immunotherapy has become an important treatment modality and significantly prolonged the survival of patients with LUAD (Reck et al., 2019; Gadgeel et al., 2020). Despite the considerable therapeutic potential, not all patients benefit equally well from immunotherapy. Therefore, it is necessary to explore the characteristics of patients who may benefit from immunotherapy and help to guide individualized treatment. Previous studies showed that TMB (Carbone et al., 2017), PD-L1 expression (Herbst et al., 2016), neoantigen (Hugo et al., 2016) might influence the efficacy of immunotherapy. Still, sometimes these markers also failed to predict the efficacy accurately. Novel markers to predict the outcomes of immunotherapy are urgently needed. Copper has been associated with cancer for more than a century, and numerous observations have suggested that tumors require higher levels of copper than healthy tissues (Blockhuys et al., 2017). Recently, Tsvetkov et al. reported a novel form of copper-dependent cell death, which is termed “cuproptosis” (Tsvetkov et al., 2022). They revealed that excess intracellular copper binds to the lipoylated components of the TCA cycle, resulting in proteotoxic stress and, ultimately, cell death. This finding provides new ideas for the application of regulating intracellular copper levels in the treatment of cancers. Additionally, the role of intracellular copper in tumor immune environments has also been found. Voli et al. reported that intracellular copper regulates key signaling pathways that mediate PD-L1-driven cancer immune evasion (Voli et al., 2020). This prompts us to test whether cuproptosis-related signatures could predict the prognosis and immunotherapy efficacy in LUAD patients.

Based on previous studies, we identified 19 CRGs involved in cuproptosis regulation. By comparing their expression levels and mutations between LUAD and normal samples, we found that these 19 CRGs are closely related to LUAD progression. Besides, the expression levels of four CRGs (*LIPT1*, *PDHA1, DLAT,* and *DLST*) are significantly correlated to the overall survival in LUAD patients. To further explore the association between these four CRGs and the immune microenvironment, we used the ESTIMATE and CIBERSORT tools to calculate the correlation of these CRGs with ImmuneScore and immune cell infiltration. Three of them (*PDHA1, DLAT,* and *DLST*) had a significant correlation with ImmuneScore, though the correlation coefficient was slightly weak. Consistent with the survival analysis results, as risk factors of LUAD, these three CRGs show a negative correlation with ImmuneScore. However, as a favorable factor of LUAD, *LIPT1* fails to correlate with ImmuneScore. *LIPT1* encodes lipoyl transferase 1, which plays an important role in mitochondrial energy metabolism (Stowe et al., 2018). Recently, *LIPT1* deficiency was demonstrated to suppress TCA cycle metabolism (Solmonson et al., 2022). Although *LIPT1* showed prognostic significance for survival in LUAD, the underlying mechanism is far to be known. In our study, we suppose that *LIPT1* may act as a tumor suppressor in LUAD by regulating other pathways rather than influencing the immune microenvironment. Besides, we were surprised to find that *DLAT* and *DLST* negatively correlate to Tregs infiltration, which is recognized as immunosuppressive cells. A recent study reported that nonresponders to PD-1 blockade therapies have a high frequency of PD-1+ effector Tregs (CD45RA^–^Foxp3^hi^CD4^+^ T cells) rather than the total of Treg cell subsets (Kumagai et al., 2020). This result suggests that different subsets of immune cells may play totally different roles in the tumor immune microenvironment. Maybe more detailed phenotyping of immune cells will lead to a more comprehensive understanding of the role of these CRGs in influencing immune cells.

According to the correlations between CRGs and immune profiles, we constructed CRG-related LUAD molecular subtypes. 122 DEGs were obtained from differential expression analysis of the two subtypes. Then, functional analyses were performed and indicated that metabolic pathways were highly enriched. Metabolic reprogramming is an important feature of tumors. Tumor cells undergo pronounced metabolic reprogramming to meet the nutrient and energy demands of rapid proliferation (Hanahan and Weinberg, 2011). As firstly described in the 1920’s, cancer cells showed enhanced glycolysis even when oxygen was abundant, which is known as the Warburg effect (Warburg, 1956). In recent years, the role of mitochondrial metabolism in tumor development has attracted more and more attention (Porporato et al., 2018). The occurrence of cuproptosis has been reported to be also closely related to mitochondrial metabolism (Tsvetkov et al., 2022; Zheng et al., 2022), which is consistent with our findings. Our study provides some clues for regulating cuproptosis by metabolic pathways.

To further understand the characteristics of cuproptosis in each patient with LUAD, we established the eight-gene cuproptosis signature (*DLST, PDHA1, FOXM1, ENO3, CD79A, AMBP, CPS1,* and *NTS*). The *α*-ketoglutarate dehydrogenase complex *DLST* encodes the enzyme responsible for the irreversible conversion of *α*-ketoglutarate into succinyl-CoA10 inside the TCA cycle, and the depletion of this enzyme impairs the growth, survival, and migration of cancer cells that have relatively intact TCA cycle function (Shen et al., 2021). As part of the pyruvate dehydrogenase enzyme complex, *PDHA1* links glycolysis with the TCA cycle and plays an important role in cancer metabolism (Liu et al., 2018). *FOXM1* is a master regulator of tumor metastasis (Raychaudhuri and Park, 2011). Deleted regions related to *ENO3* are involved in lung cancer (Tsuchiya et al., 2000). *CD79A* is altered in many cancers, including lung cancer (2017). *ABMP* may play a role in the regulation of inflammatory processes. *CPS1* is the rate-limiting mitochondrial enzyme in the urea cycle (Wu et al., 2020). *NTS* are autocrine growth factors that are secreted by tumor cells and bind to receptors on the cell surface to stimulate tumor growth (Moody et al., 2018). As expected, a higher cuproptosis score predicted a poor prognosis in LUAD. When checking the somatic mutation frequencies, we found *TP53*, *TTN*, and *SPTA1* were significantly different between high and low cuproptosis score groups. *TP53* mutation profoundly affects tumor cell genomic structure and expression (Donehower et al., 2019). *TTN* is a key component in muscle assembly and function and plays a role in chromosome condensation and chromosome segregation during mitosis (Mayans et al., 1998; Itoh-Satoh et al., 2002). *SPTA1* is the major constituent of the cytoskeletal network underlying the erythrocyte plasma membrane. And interestingly, *TP53* mutation is reported to be associated with poor prognosis of lung cancer patients (Ma et al., 2016). We did not find the prognostic significance of *TP53* mutation in our study. Only the cuproptosis score suggests a significant correlation with LUAD prognosis in the univariate Cox regression analysis, which demonstrated the independent predictive power of the cuproptosis score.

We also analyzed the characteristics of the immune microenvironment between the high and low cuproptosis score groups. The expression of some immune cells, such as activated B cells, activated CD8^+^ T cells, activated dendritic cells, and T helper cells, was significantly higher in the low cuproptosis score group. This finding indicates that with a decrease in the cuproptosis score, the antitumor immune effect was promoted. B cells can present tumor-associated antigens to T cells or produce antibodies that increase antigen presentation to T cells or kill tumor cells (Fridman et al., 2021). CD8^+^ T cells are the most powerful effectors in the anticancer immune response (Raskov et al., 2021). Dendritic cells are central regulators of the adaptive immune response and are necessary for T-cell-mediated cancer immunity (Gardner and Ruffell, 2016). Thus, a low cuproptosis score is associated with activated antitumor immunity. In addition, the stemness score (RNAss) was positively associated with the cuproptosis score. Cancer stem cells are capable of self-renewal, differentiation, and proliferation, which are responsible for the reconstitution and propagation of the disease (Prasad et al., 2020). Additionally, cancer stem cells influence on immune cells, including tumor-associated macrophages, myeloid-derived suppressor cells, and T cells (Chen et al., 2021). The higher the cuproptosis score is, the stronger the heterogeneity of the tumor and the worse the prognosis.

We further demonstrated the predictive power of the cuproptosis score for immunotherapy. In our study, 34 patients of LUAD treated with anti-PD-1 were divided into high and low cuproptosis score groups. The ORR was much lower in the high cuproptosis score group. What’s more, patients with high cuproptosis scores tended to be distributed in the nonresponder group. Although these differences were not statistically significant, the limited sample sizes might bias the result. We will expand the sample size in future studies to verify our findings.

Our study is the first to establish a prognostic model in LUAD patients based on cuproptosis and immune profiles. This model integrated gene features, clinical information (sex, age, T stage, and N stage) of LUAD patients, and the cuproptosis score, which helped predict the survival probability of LUAD at 1, 3, and 5 years. Internal and external validation demonstrated the good predictive value of this model, and the cuproptosis score itself can be used as an independent prognostic factor for LUAD patients. These findings may advance our understanding of the relationship between cuproptosis and the immune microenvironment, which will help predict the prognosis and efficacy of immunotherapy in LUAD.

This study has some limitations. A large number of LUAD samples were needed to verify the stability of the typing. To date, the number of identified CRGs is still small, and our study also relied on the correlation between CRGs and the ImmuneScore. The relationship between cuproptosis and immunity requires further experimental verification.

## 5 Conclusion

In summary, we identified novel cuproptosis subtypes based on CRGs and immune profiles, providing insight into cuproptosis in LUAD. Additionally, we evaluated the underlying mechanisms of the cuproptosis subtypes, including the characteristics of the TME, metabolic processes, and multi-omics properties. Finally, we developed a new cuproptosis score for individual tumors, which may advance our understanding of the relationship between cuproptosis and the immune microenvironment. The cuproptosis signature based on the cuproptosis score and clinical characteristics of individual patients will be useful for guiding immunotherapy in LUAD.

## Data Availability

The original contributions presented in the study are included in the article/Supplementary Material, further inquiries can be directed to the corresponding authors.

## Supplementary material

Supplementary Figure S1 Univariate Cox regression of prognosis and 3 genes that are significantly different in high and low cuprotosis score groups.

Supplementary Table S1 Mutation frequency of top 20 genes in high and low cuprotosis score groups.
