# Supplementary material for: Molecular subtypes based on cuproptosis-related genes and immune profiles in lung adenocarcinoma
Source: Front Genet. 2022 Oct 12;13:1006938. doi: 10.3389/fgene.2022.1006938 (PMC9597639; doi:10.3389/fgene.2022.1006938)
Supplement: Supplementary file 1 [file Table1.DOCX]

| Genes | Type | Cuproptosis_score high (N=262) | Cuproptosis_score low (N=234) | Fisher‘s exact test p-value |
| --- | --- | --- | --- | --- |
| TP53 | Number of patients with mutations | 134 | 94 | 0.015 |
|  | Number of patients without mutations | 128 | 140 |  |
| TTN | Number of patients with mutations | 126 | 89 | 0.029 |
|  | Number of patients without mutations | 136 | 145 |  |
| MUC16 | Number of patients with mutations | 110 | 89 | 0.409 |
|  | Number of patients without mutations | 152 | 145 |  |
| CSMD3 | Number of patients with mutations | 100 | 89 | 1 |
|  | Number of patients without mutations | 162 | 145 |  |
| RYR2 | Number of patients with mutations | 97 | 80 | 0.573 |
|  | Number of patients without mutations | 165 | 154 |  |
| LRP1B | Number of patients with mutations | 84 | 75 | 1 |
|  | Number of patients without mutations | 178 | 159 |  |
| ZFHX4 | Number of patients with mutations | 81 | 75 | 0.846 |
|  | Number of patients without mutations | 181 | 159 |  |
| USH2A | Number of patients with mutations | 86 | 63 | 0.17 |
|  | Number of patients without mutations | 176 | 171 |  |
| KRAS | Number of patients with mutations | 76 | 61 | 0.483 |
|  | Number of patients without mutations | 186 | 173 |  |
| XIRP2 | Number of patients with mutations | 63 | 51 | 0.594 |
|  | Number of patients without mutations | 199 | 183 |  |
| FLG | Number of patients with mutations | 58 | 56 | 0.67 |
|  | Number of patients without mutations | 204 | 178 |  |
| SPTA1 | Number of patients with mutations | 68 | 42 | 0.039 |
|  | Number of patients without mutations | 194 | 192 |  |
| NAV3 | Number of patients with mutations | 55 | 44 | 0.575 |
|  | Number of patients without mutations | 207 | 190 |  |
| ZNF536 | Number of patients with mutations | 52 | 44 | 0.82 |
|  | Number of patients without mutations | 210 | 190 |  |
| COL11A1 | Number of patients with mutations | 50 | 47 | 0.821 |
|  | Number of patients without mutations | 212 | 187 |  |
| ANK2 | Number of patients with mutations | 47 | 44 | 0.817 |
|  | Number of patients without mutations | 215 | 190 |  |
| FAT3 | Number of patients with mutations | 50 | 40 | 0.641 |
|  | Number of patients without mutations | 212 | 194 |  |
| PCLO | Number of patients with mutations | 47 | 42 | 1 |
|  | Number of patients without mutations | 215 | 192 |  |
| CSMD1 | Number of patients with mutations | 47 | 42 | 1 |
|  | Number of patients without mutations | 215 | 192 |  |
| APO8 | Number of patients with mutations | 50 | 37 | 0.347 |
|  | Number of patients without mutations | 212 | 197 |  |

**Table 1. Mutation frequency of top 20 genes in high and low cuprotosis score groups.**
