# Supplementary figures and images for: Molecular subtypes based on cuproptosis-related genes and immune profiles in lung adenocarcinoma
Source: Front Genet. 2022 Oct 12;13:1006938. doi: 10.3389/fgene.2022.1006938 (PMC9597639; doi:10.3389/fgene.2022.1006938)

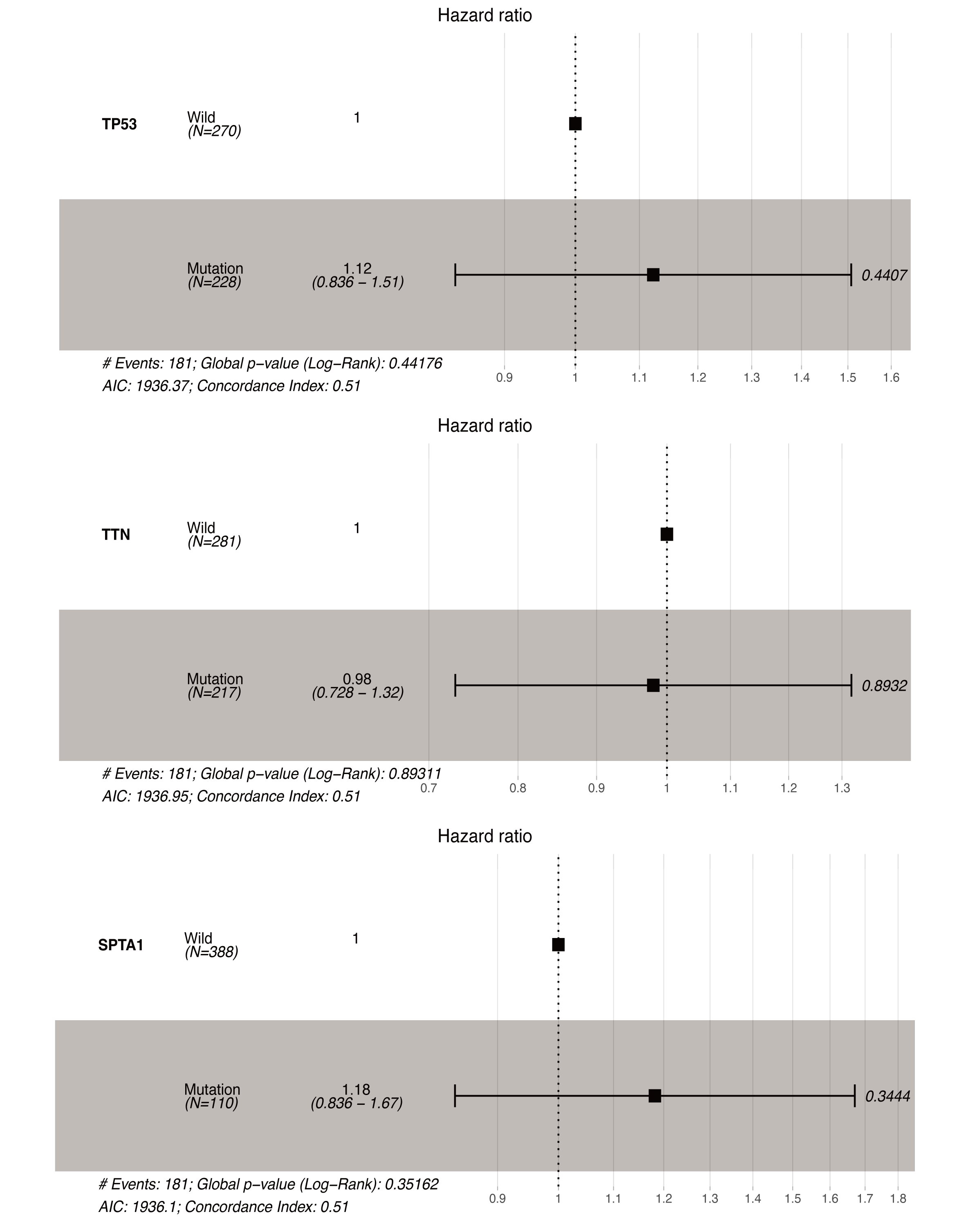

Supplement: Supplementary file 2 [file Image1.TIF]
